# Optical Genome Mapping Helps to Identify *BCR::JAK2* Rearrangement Arising from Cryptic Complex Chromosomal Aberrations: A Case Report and Literature Review

**DOI:** 10.3390/genes14122188

**Published:** 2023-12-08

**Authors:** Neelam Vanjari, Guilin Tang, Gokce A. Toruner, Wei Wang, Beenu Thakral, Ming Zhao, Bhavana J. Dave, Joseph D. Khoury, L. Jeffrey Medeiros, Zhenya Tang

**Affiliations:** 1 School of Health Professions, The University of Texas MD Anderson Cancer Center, Houston, TX 77015, USA; vanjarn@labcorp.com (N.V.);; 2 Department of Hematopathology, The University of Texas MD Anderson Cancer Center, Houston, TX 77015, USAbthakral@mdanderson.org (B.T.); ljmedeiros@mdanderson.org (L.J.M.); 3 Department of Pathology, Microbiology and Immunology, University of Nebraska Medical Center, Omaha, NE 68198, USA

**Keywords:** *BCR::JAK2* rearrangement, optical genome mapping (OGM), complex chromosomal aberrations, myeloproliferative neoplasm (MPN), fluorescence in situ hybridization (FISH)

## Abstract

We report a case of myeloproliferative neoplasm, not otherwise specified (MPN-NOS)-transformed AML with *BCR::JAK2* rearrangement. Chromosomal analysis indicated a simple abnormal karyotype 46,XY,t(7;17)(q21;q24),t(9;22)(p24;q11.2). Fluorescence in situ hybridization (FISH) using a BCR/ABL1/ASS1 probe set suggested a possible *BCR* rearrangement and a reflex JAK2 breakapart probe indicated *JAK2* rearrangement, most likely partnered with *BCR*. Optical genome mapping (OGM) analysis confirmed *BCR::JAK2* derived through an inv(9)(p24p13) after a t(9;22)(p13;q11.2) in this case. Due to the complexity of chromosomal aberrations, disruption and/or rearrangement of other genes such as *KIF24::BCR*, *JAK2::KIF24/UBAP1,* and *CDK6:SOX9* were also identified by OGM. Although the functionality and clinical importance of these novel rearrangements were unknown, disruption of these genes might be associated with a poorer response to chemotherapy and disease progression. We also reviewed all cases with *BCR::JAK2* rearrangement reported in the literature. In conclusion, a suspected t(9;22)/*BCR::JAK2* rearrangement warrants further characterization with genomic assays such as OGM, whole chromosome sequencing, and RNA sequencing to explore other gene disruptions and/or rearrangements.

## 1. Introduction

Rearrangement between *BCR*, located at chromosome 22q11.2, and *JAK2,* located at chromosome 9p24.1 (*BCR::JAK2*), is a recurrent but infrequent chromosomal aberration. To date, 17 cases with confirmed *BCR::JAK2* rearrangement have been reported in the literature, mostly in patients with myeloproliferative neoplasms, not otherwise specified (MPN-NOS) [1,2,3,4,5,6,7,8,9,10,11], but also in patients with acute myeloid leukemia (AML) [12], acute B-lymphoblastic leukemia (B-ALL) [13,14,15,16], and myeloid/lymphoid neoplasms with eosinophilia [10,17]. In some cases, the AML or B-ALL has transformed from an antecedent MPN [10,15,16]. At least from the chromosomal analysis results described in their reports, a simple and balanced t(9;22)(p24;q11.2)/*BCR::JAK2* has been reported in most cases [1,2,4,6,7,12,16]. A three-way translocation, involving one more chromosome other than chromosomes 9 and 22, has been reported in some cases and most of these also showed a balanced three-way translocation [3,11,14,15]. An insertion, apparent ins(22;9)(q11;p24p13) [5] or cryptic ins(22;9)(q11;p24p24) [10], has been reported in two cases, whereas the underlying chromosomal aberration(s) related to *BCR::JAK2* were not fully characterized and described in other cases [9,13,17]. 

Since both *JAK2* and *BCR* are in an orientation of plus strand on chromosomal loci 9p and 22q, respectively, a simple, reciprocal t(9;22)(p24;q11.2) will generate a head-to-head (*5′BCR::5′JAK2*) and a tail-to-tail (*3′BCR::3′JAK2*) recombination that are insufficient to form an in-frame, functional *3′BCR::5′JAK2* or *BCR::JAK2* fusion. Therefore, in those cases with morphologically balanced t(9;22) and/or three-way translocations, the *BCR::JAK2* rearrangement must have been derived from a complex genomic rearrangement, such as translocation plus inversion, which might not be totally detected by standard chromosomal analysis. Here, we report a new *BCR::JAK2* rearranged case that arose from complex aberrations involving chromosomes 9 and 22. Intensive FISH studies and the novel technology of optical genome mapping (OGM) analysis were applied to confirm *BCR::JAK2* rearrangement and further characterize genome-wide structural variants (SVs) and copy number variants (CNVs) in this case. OGM analysis confirmed all findings detected by both chromosomal and FISH analyses and revealed several novel rearrangements/fusions which may be associated with disease progression.

## 2. Case Report

A 39-year-old man presented with a myeloproliferative/myelodysplastic syndrome (MPN/MDS) diagnosed at an outside institution. According to the submitted reports, the initial bone marrow examination showed 95% hypercellularity with grade I myelofibrosis without any increase in blasts. Conventional cytogenetic analysis of bone marrow (BM) showed 46,XY in six cells and then, in a subsequent specimen, t(7;17)(q22;q25),t(9;22)(p13;q11.2). BCR/ABL Dual Fusion (DF) FISH analysis showed a negative result for *BCR::ABL1* rearrangement but did detect an extra *BCR* signal in 76% of analyzed cells. The patient received chemotherapy and ruxolitinib for 3 months but developed disease progression. He was then referred to our hospital in December 2022. 

**Figure 1:**
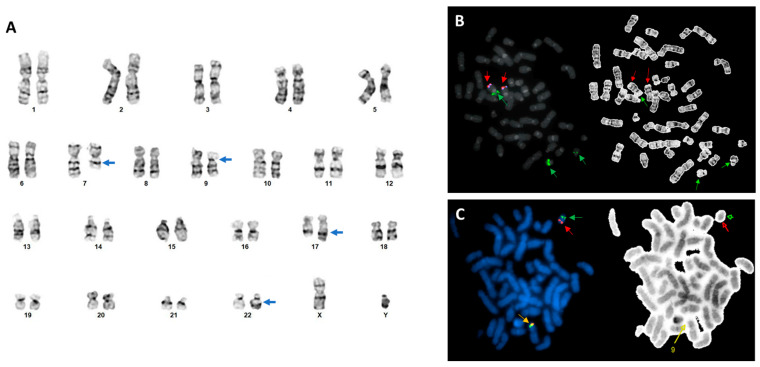
Chromosomal and FISH analyses in this case. (**A**) A representative karyogram with abnormal chromosomes 7, 9, 17, and 22 pointed out by arrows. (**B**) Representative BCR/ABL1/ASS1 metaphase FISH (*left*) and inverted (*right*) images. Red arrows: ABL1/ASS1 probe; Green: BCR probe. The der(9) has an ABL1/ASS1 (red) signal on its q arm and a BCR (green) signal on its p arm, while the der(22) has a dim BCR (green) signal on it. (**C**) Representative JAK2 BAK metaphase FISH (*left*) and inverted (*right*) images. Red arrows: *5′JAK2* signal; Green: *3′JAK2* signal probe; Orange: intact *JAK2* signal. The normal 9 has an intact JAK2 signal on it, while the der(22) has the split-apart *5′JAK2* (red) and *3′JAK2* (green) signals on it.

Bone marrow aspiration and biopsy showed acute myeloid leukemia (AML) with megakaryoblast differentiation and 25% blasts. The blasts were CD33+ and CD123+. Conventional chromosomal analysis revealed morphologically balanced t(7;17)(q21;q24) and t(9;22)(p24;q11.2) observed in all 20 cells analyzed (Figure 1A), and the karyotype was initially proposed as 46,XY,t(7;17)(q21;q24),t(9;22)(p24;q11.2) [18]. The Vysis BCR/ABL1/ASS1 Tri-Color DF FISH Probe (Abbott Molecular, Abbott Park, IL) was employed and did not detect *BCR::ABL1* rearrangement, but an additional *BCR* signal was detected in all 200 cells analyzed (100%) (Figure 1B). Interestingly, one *BCR* (green) signal was located on the short (p) arm of the abnormal chromosome 9, der(9), and was very close to the centromere of der(9) as well, whereas a dim green signal was located on the der(22), indicating a gain of an additional green signal likely due to a *BCR* rearrangement. Vysis 5p15.2/5q35, CEP7/7q31, and TP53/CEP17 FISH tests were negative. Therefore, the CytoCell JAK2 breakapart FISH (OGT Inc., Tarrytown, NY, USA) was then reflexed with a positive result for *JAK2* rearrangement (Figure 1C) in all 200 cells analyzed (100%). Both the split-apart *5′JAK2* (red) and *3′JAK2* (green) signals were located on the der(22), and the *3′JAK2* (green) was close to the centromere of the der(22), almost at the same place as the dim *BCR* signal mentioned above. This result suggested a *BCR::JAK2* rearrangement, whereas the *5′JAK2* (red) signal was close to the telomere of the der(22) (Figure 1C). Taken together, a *BCR::JAK2* rearrangement was postulated to have formed through two steps of chromosomal recombination: a t(9;22)(p13;q11.2) causing the *BCR* rearrangement suggested by BCR/ABL1/ASS1 FISH and an inversion of 9p on the der(22), inv(9)(p13p24), driving the *JAK2* rearrangement confirmed by JAK2 BAP FISH, as well as very likely a *BCR::JAK2* rearrangement that was later further confirmed by optical genome mapping (OGM) (see below). Therefore, the final chromosomal analysis result was reported as the following: 46,XY,t(7;17)(q21;q24),der(9)t(9;22)(p13;q11.2),der(22)t(9;22)inv(9)(p13p24) [20] by following the ISCN 2020 [18].

**Figure 2:**
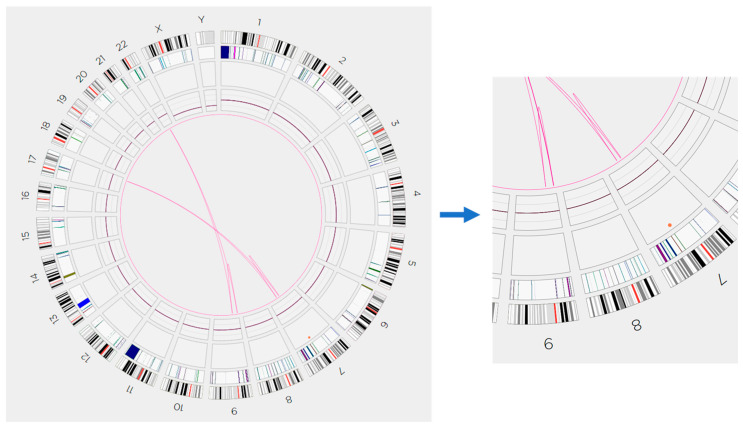
The OGM analysis in this case. (***Left***) OGM Circos plot illustrates the chromosomal aberrations in this case. (***Right***) The enlarged views of the areas containing chromosomes 7 to 9 indicated two types of t(7;17)s, two types of t(9;22), and intra-chromosomal fusions involving chromosomes 7 and 9, respectively. Please see detailed information of these chromosomal aberrations in Table 1 and Table 2.

**Figure 3:**
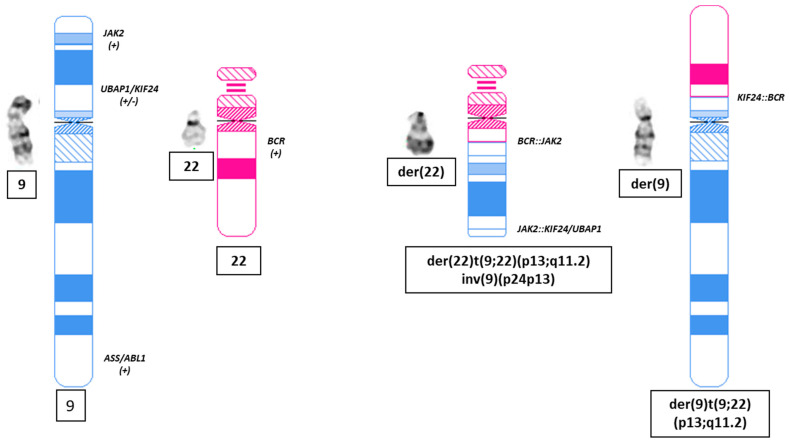
Illustration of the aberrations and locations of affected genes on chromosomes 9 (blue color) and 22 (red color). (***Left***) Normal chromosomes 9 and 22 and locations of certain genes on them. (***Right***) der(22) and der(9) and locations of certain gene rearrangements on them. der: derivative.

Ultra-high molecular weight genomic DNA extracted from the bone marrow aspirate specimen was subjected to direct labeling and staining (DLS) and was then assessed by OGM, as reported previously [19]. OGM analysis detected co-existence of t(9;22)(p24.1;q11.23)/*BCR::JAK2*, t(9;22)(p13.3;q11.23)/*KIF24::BCR* and an intra-chromosomal fusion between 9p24.1 and 9p13.3 with a *JAK2::KIF24/UBAP1* (Figure 2 and Table 1). In general, an intra-chromosomal fusion detected by OGM can be theoretically caused by any recombination between two chromosomal homologs (such as translocation and insertion) or an intra-homolog recombination (such as inversion, segmental deletion, and/or duplication). Both *KIF24* and *UBAP1* are located at 9p13.3, and they are adjacent to each other with a small overlap of approximately 140 base pairs (bp). Interestingly, OGM also detected t(7;17)(21.13;q24) without any gene fusion, t(7;17)(21.2;q24)/*CDK6:SOX9*, and an intra-chromosomal fusion between 7q21.13 and 7q21.2 via deletion of 7q21.13q21.2 of approximately 3 Mbp or del(7)(q21.13q21.2)(chr7: 89,828,122–92,814,090) (Figure 2 and Table 2). In summary, in correlation with chromosomal and FISH analysis findings, OGM analysis showed that der(9) was formed through t(9;22)(p13.3;q11.23) with *KIF24::BCR* rearrangement and der(22) was derived from t(9;22) followed by an inv(9)(p24p13.3) with *BCR::JAK2* rearrangement on the centromeric side and *JAK2::KIF24/UBAP1* rearrangement on the telomeric side, as illustrated in Figure 3. The t(7;17)(21.13;q24) and the t(7;17)(21.2;q24) cannot be distinguished by conventional chromosomal analysis at the current level of resolution (<550 band level), and the microdeletion of 7q21.13q21.2 causing these translocations involving two different band levels of 7q is also cryptic for chromosomal analysis. The patient was treated with cladribine and cytarabine in our hospital, but his disease progressed rapidly and he died 3 months later. At his last workup, JAK2 BAP FISH was still positive (33%), but chromosomal analysis could not be repeated due to a hypocellular bone marrow specimen.

## 3. Discussion

Although *BCR::JAK2* rearrangement is relatively rare in MPNs, detection/confirmation of this mutation may qualify affected patients for targeted therapy, such as ruxolitilib alone [13,14] and/or in a combination with BCL2 inhibitors such as venetoclax [11]. To the best of our knowledge, there are 17 cases with confirmed *BCR::JAK2* rearrangement in the literature, with an additional 5 cases with t(9;22)(p24;q11.2) and suspicious *BCR::JAK2* rearrangement solely based on karyotype only and/or limited FISH studies (Table 3) [20,21,22,23,24]. Interestingly, 4 of the 5 cases suspicious for *BCR::JAK2* rearrangement also showed a morphologically balanced t(9;22)(p24;q11.2). In fact, the B-ALL case reported by Tirado et al. [20] had a *JAK2* rearrangement due to an additional *JAK2* signal located on the der(22), but the authors could not prove a simultaneous *BCR* rearrangement or a *BCR::JAK2* fusion by intensive FISH studies. Chen et al. [24] also reported a B-ALL case with t(9;22)(p24;q11.2). However, they failed to detect either *JAK2::BCR* or *BCR::JAK2* fusion transcripts in that case. By adding the case with *BCR::JAK2* confirmed by intensive FISH and OGM analyses in this study, the database of *BCR::JAK2-*positive cases has been increased to a total of 18 cases. This is also the second *BCR::JAK2-*positive AML case transformed from a MPN-NOS [12] (Table 3).

*BCR::JAK2* rearrangement via an insertion of *3′JAK2* into the *BCR*, either as an apparent [5] or as a cryptic [10] chromosomal aberration, has been reported in two cases. An insertion of the *5′BCR* into the *ABL1* is also theoretically capable of generating a functional *BCR::JAK2* rearrangement, but no such cases like this have been reported. However, for a t(9;22)(p24;11.2) with suspicious *BCR::JAK2* rearrangement to occur, as demonstrated in our case, a complex chromosomal recombination must have brought *BCR* and *JAK2* into juxtaposition to generate a pathogenic *5′BCR::3′JAK2* or *BCR::JAK2* fusion. Therefore, the t(9;22)(p24;11.2) and other three-way translocations involving 9p24 and 22q11.2 in the previously reported cases were most likely described solely according to the morphology of chromosomal aberrations. It is relevant to mention that *BCR::JAK2* fusions have been confirmed by RT-PCR, Sanger Sequencing, and/or other methods in the majority of these cases (Table 3). Interestingly, several isoforms of *BCR::JAK2* fusion transcripts have been isolated, e.g., *BCR::JAK2/E1::E17* (6 cases), *BCR::JAK2/E1::E19* (5 cases), *BCR::JAK2/E1::E15* (3 cases), *BCR::JAK2/E14::E11* (1 case), and *BCR::JAK2/E13::E17* (1 case). Co-existence of two isoforms has been observed in 3 cases. The clinical significance of these isoforms remains unknown. For our case reported in this study, a *BCR::JAK2/E1::E17* (Table 1) is postulated by applying the breakpoints of *5′BCR* and *3′JAK2* obtained by OGM into the UCSC Genome Browser, respectively.

The complexity of chromosomal aberrations usually indicates the possibility of mutations simultaneously involving multiple genes, in addition to *BCR* and *JAK2*. Although chromosomal analysis was reported as a simple abnormal karyotype with t(9;22)(p24;q11.2) in a B-ALL case, Chen et al. [16] showed a co-existence of *JAK2::PPM1F* rearrangement and *PRAMENP::BCR* rearrangement by whole genome sequencing (WGS). A novel *JAK2::PPM1F/E16::E2* fusion was further identified by RT-PCR and Sanger sequencing, although it was postulated as non-functional due to induction of a stop codon in the fusion gene. In a myeloid/lymphoid neoplasm with eosinophilia case reported by Snider et al. [10], as further characterized by mate-pair sequencing, involvement of four derivative chromosomes (der(8), der(9), and two copies of der(22)) to form the *BCR::JAK2* rearrangement and a novel *PCM::JAK2/E7::E9E10* rearrangement were identified. In the case we report here, the co-existing *KIF24::BCR* and *JAK2::KIF24/UBAP1* rearrangements were detected by OGM. Although a functional fusion transcript from these two novel rearrangements cannot be determined, *KIF24* and/or *UBAP1* were certainly disrupted in our case.

As a novel technology, OGM has played an important role in further characterizing cryptic chromosomal aberrations and confirming *BCR::JAK2* rearrangement. OGM analysis also revealed various novel rearrangements such as *KIF24::BCR*, *JAK2::KIF24/UBAP1*, and *CDK6:SOX9* and microdeletion of del(7)(q21.13q21.2) in the case we reported. All these novel findings were not detected by routine chromosomal analysis and targeted FISH, and these abnormalities might be associated with a poor response to chemotherapy and disease progression from MPN-NOS to AML, as occurred in this patient. As suggested by Snider and others, all cases with confirmed and/suspected *BCR::JAK2* rearrangement warrant analysis using integrated genomic approaches such as WGS, RNA sequencing, and OGM to fully characterize the underlying complex chromosomal aberrations.

In summary, cases with *BCR::JAK2* rearrangement are infrequent and chromosomal aberrations producing a functional *BCR::JAK2* fusion can be complicated. Very likely, disruption and/or rearrangement of other important gene(s) can be co-existent. Utilizing integrated genomic approaches such as OGM, WGS, and/or RNA sequencing is strongly recommended.

## Figures and Tables

**Table 1:** The detailed information of aberrations involving chromosome 9 and 22, including breakpoints, confidence, VAF, putative gene fusion, and ISCN called by using Bionano Access Software (v1.7) (the Genome Reference Consortium Human Reference 38 (GRCh38) has been applied).

SV Type	Chr Involved #1	Chr Involved #2	Breakpoint #1 (bp)	Breakpoint #1 (bp)	Confidence	VAF	Putative Gene Fusion	ISCN
Transl.	9	22	34,271,272	23,219,177	0.98	0.48	KIF24-BCR	ogm[GRCh38) t(9;22)(p13.3;q11.23)
Transl.	9	22	5,079,902	23,205,817	0.99	0.5	JAK2-BCR	ogm[GRCh38) t(9;22)(p24.1;q11.23)
intrachr_fusion	9	22	5,077,416	34,246,297	0.99	0.47	JAK2-UBAP1/KIF24	ogm[GRCh38) fus(9;9)(p24.1;p13.3)

SV: structural variant; Chr: chromosome; bp: base pair; Transl.: translocation; intrachr_fusion: intra-chromosomal fusion; fus: fusion; VAF: variant allele frequency.

**Table 2:** The detailed information of aberrations involving chromosomes 7 and 17 detected by OGM analysis (the Genome Reference Consortium Human Reference 38 (GRCh38) has been applied).

SV Type	Chr Involved #1	Chr Involved #2	Breakpoint #1 (bp)	Breakpoint #1 (bp)	Confidence	VAF	Putative Gene Fusion	ISCN
Transl.	7	17	108,464,953	72,155,558	1	0.45		ogm[GRCh38) t(7;17)(q31.1;q24.3)
Transl.	7	17	92,814,090	72,155,558	1	0.38	CDK6-SOX9	ogm[GRCh38) t(7;17)(q21.2;q24.3)
intrachr_fusion	7	7	89,954,704	108,465,844	0.94	0.45		ogm[GRCh38) fus(7;7)(q21.13;q31.1)

SV: structural variant; Chr: chromosome; bp: base pair; Transl.: translocation; intrachr_fusion: intra-chromosomal fusion; fus: fusion; VAF: variant allele frequency.

**Table 3:** Twenty-three cases with confirmed and suspicious *BCR::JAK2* rearrangement reported in the literature, including the case reported in this study.

Year	Authors	Age/Sex	Diagnosis	Chromosomal Analysis	FISH Analysis	Confirmation of *BCR::JAK2*	BCR::ABL1 Isoform(s)
2005	Griesinger et al. [1]	63 yo, F	MPN-NOS	46,XX,del(7)(q22q36)[21]/47,idem,t(9;22)(p24;q11.2),+19[5]	BCRx3 by BCR/ABL1 DF FISH	Yes. RT-PCR and Sanger Seq	E1::E19
2008	Cirmena et al. [12]	67 yo, F	AML *	46,XX,t(9;22)(p24;q11)	BCRx3 by BCR/ABL1 DF FISH	Yes. RT-PCR and Sanger Seq	E14::E11
2008	Lane et al. [2]	44 yo, M	MPN-NOS	46,XY,t(9;22)(p24;q11.2) [23]/46,XY[27]	BCR/ABL1 DF FISH-negative	Yes. RT-PCR and Sanger Seq	E1::E17
2011	Impera et al. [3]	54 yo, F	MPN-NOS	46,XX,t(9;18;22)(p23;p11.3;q11.2)[18]/46,XX[2]	BCR/ABL1 FISH-negative, other intensive FISH studies	Yes. SNP array, RT-PCR, and Sanger Seq	E1::E15 and E1::E17
2012	Cuesta-Dominguez et al. [13]	55 yo, F	B-ALL	49,XY,+X,+2,+4,−9,−11,+19,add(19)(q13),+20,−22,+mar[24]/46,XY[1]	BCR/JAK2 fusion FISH-positive	Yes. RT-PCR and Sanger seq	E1::E19
2012	Elnaggar et al. [4]	84 yo, M	MPN-NOS	46,XX,t(9;22)(p24;q11.2)	BCRx3 by BCR/ABL1 DF FISH	Yes. RT-PCR and Sanger seq	E1::E19
2012	Roberts et al. [14]	2 yo, M	B-ALL	47,XY,+2,del(2)(p23),t(3;22;9)(p12;q11.2; p24)[10]/46,XY[2]	n/a	Yes. WGS, RNA Seq, RT-PCR, and Sanger Seq	E1::E15 and E1::E17
2013	Xu et al. [5]	28 yo, M	MPN-NOS	ins(22;9)(q11;p13p24)	JAK2 BAP-positive	Yes. RT-PCR and Sanger seq	E1::E19
2015	Chamseddine et al. [6]	49 yo, M	MPN-NOS	46,Y,t(9;22)(p24;q11)[12/20]/47,idem,+der(22)t(9;22)[2/20]/46,XY [6/20]	BCRx3 by BCR/ABL1 DF FISH; JAK2 BAP FISH-positive	Yes. RT-PCR and Sanger seq	E13::E17
2015	Schwaab et al. [7]	?	MPN-NOS	t(9;18)(p24;q12),der(18)ins(22)(BCR)	JAK2 BAP-positive	Yes. RT-PCR and Sanger seq	E1::E17
2016	Duployez et al. [15]	58 yo, M	B-ALL *	47,XY,+8,t(9;22;15)(p24;q11;q21)(at MPN-U stage)	JAK2 BAP FISH-positive	Yes. RT-PCR and Sanger seq	E1::E17
2016	He et al. [8]	36 yo, F	MPN-NOS	Complex karyotype with multiple rearrangements involving chromosome 22, including a non-classical, unbalanced t(9;22) resulting in a derivative 9 from a t(9;22) and a dicentric rearrangement involving chromosomes 22 and 10.	BCRx3 by BCR/ABL1 DF FISH; JAK2 BAP FISH-positive	Yes. RT-PCR and Sanger seq	E1::E15 and E1::E17
2019	Millett et al. [9]	53 yo, M	MPN-NOS **	n/a	JAK2 BAP-positive	Yes. RT-PCR	n/a
2020	Snider et al. [10]	59 yo, M	Myeloid/lymphoid neoplasm with eosinophilia	46,XY,t(3;21)(q21;q22)[10]/47,XY,+11[5]/46,XY[5]	JAK2 BAP-positive	Yes. Mate-pair seq	n/a
2020	Thakral et al. [17]	31 yo, M	Myeloid neoplasm with eosinophilia	46,XY,add(22)(q11.2)[13]/46,XY[7]	BCRx3 by BCR/ABL1 DF FISH	Yes. RNA seq	n/a
2021	Chen et al. [16]	32 yo, M	B-ALL *	46,XY,t(9;22)(p24;q11.2)[15]/46,XY[5] (with IKZF1 deletion)	BCR signal on der(9) by BCR/ABL1 DF FISH	Yes. WGS, RT-PCR, and Sanger Seq	E1::E19
2021	Lap et al. [11]	54 yo, M	MPN-NOS **	46,XY,del(6)(p21.2p24),t(22;9;11)(q11.2,p24,p11.2)[11]/46XY,del(9)[9]	BCRx3 by BCR/ABL1 DF FISH; JAK2 BAP FISH-positive	Yes. RT-PCR and Sanger seq	E1::E19
2010	Tirado et al. [20]	14 yo, M	B-ALL	46,XY,t(9;22)(p24;q11.2)[14]/46,XY[6]	JAK2 BAP-positive	No	
2011	Angelova et al. [21]	53 yo, M	MPN-NOS/MDS with Eos and baso	47,XY,dup(3)(q25q27),+8,der(9)t(9;22)(p24;q11),dup(14)(q24q32),+14,−22[13]/47,XY,dup(1)(p13p22),dup(3)(q25q27),+8,add(8)(q24),der(9)t(9;22)(p24;q11),−22,+mar [6]/47,XY,dup(3)(q25q27),+8,t(9;22)(p24;q11),dup(14)(q24q32),+14,−21[1]	No	No	n/a
2013	Bellesso et al. [22]	54 yo, M	MPN-NOS	46,XY,t(9;22)(p24;q11.2)	BCR/ABL1 DF, BCR/PDGFR DF FISH-negative	No	n/a
2015	Kantarcioglu et al. [23]	64 yo, F	MDS	47,X,-X,+3,t(9;22)(p24;q11.2),+18[3]/46,XX[17]	BCR/ABL1 DF	No	
2020	Chen et al. [24]	45 yo, M	B-ALL	46,XY,t(9;22)(p24;q11.2)[9]/46,XY[2]	No	No JAK2-BCR and BCR-JAK2 fusion was detected by RT-PCR	
This study	Vanjari et al.	39 yo, M	AML *	46,XY,t(7;17)(q21;q24),der(9)t(9;22)(p13;q11.2),der(22)t(9;22)inv(9)(p13p24)[20]	BCRx3 by BCR/ABL1/ASS1 DF FISH; JAK2 BAP FISH-positive	Yes. OGM	E1:E17

* Transformed from previous MPN-NOS. ** Previous history of HL and chemotherapy.

## Data Availability

Data are contained within the article.
